# Enhancing Transformer-Based Language Models for Hungarian Handwritten Text Recognition

**DOI:** 10.12688/f1000research.176408.2

**Published:** 2026-03-19

**Authors:** Mohammed A.S. Al-Hitawi, Natabara Máté Gyöngyössy

**Affiliations:** 1 Department of Artificial Intelligence, College of Information Technology, University of Fallujah, 31002 Fallujah, Iraq; 2 Department of Artificial Intelligence, Faculty of Informatics, Eötvös Loránd University (ELTE), 1117 Budapest, Hungary

**Keywords:** Deep Learning, Cultural Heritage, Handwriting Text Recognition (HTR), Image-to-Text (I2T), Language Models, Natural Language Processing, Optical Character Recognition (OCR), Pattern Recognition, Scene Text Recognition (STR), Self-attention, Sequence-to-Sequence

## Abstract

Optical Character Recognition (OCR) is still working on making a multilingual model that incorporates the Hungarian language. We introduce a hybrid Hungarian and English model, one of the biggest challenges is to recognize handwritten text. We are going to investigate a set of models in this research, such as TrOCR large-handwritten, leveraging PULI-BERT, and Roberta-base with Diet models. The digitization of documents, and the preservation of cultural heritage specifically, has long been a research problem related to text recognition. We use an extensive text on the recognition approach using pre-trained visual and language transformer models. We pre-train the TrOCR proposed by Microsoft researchers for both large and base models at the first phase and then fine-tune them on human data at the second stage. Then, leverage new pre-trained transformers models such as Roberta-base, and PULI-BERT, as decoders and Diet, Vit, and Beit as encoder models at the pre-training phase on generated synthetic data and then fine-tune them on a small amount of human-annotated data provided by (DH-Lab) researchers with augmentation and without augmentation. Developed using tiny-scale Synthetic data of around three-million-line text open-source corpus, and subsequently refined using tiny person-labeled datasets.

Experiments showed that the best CER is 3.681 in the TrOCR large handwritten, and the best WER is 16.091 by leveraging the PULI-BERT with the Deit model. These fine-tuned models outperform the currently existing state-of-the-art TrOCR models on historical Hungarian handwriting, according to the benchmark results on the János Arany dataset.

## Introduction

OCR has been performed very well for the English language, but there are some limitations and high error rates for non-English languages such as Arabic due to mixed letters in words (there is no space for most of the letters) and Hungarian has some special characters; therefore, we are going to address the gap for offline handwriting recognition for the Hungarian language, which is the most common deep-learning problem. As Hungarian has a distinctive alphabet and handwriting style, OCR software made specifically for this language may perform better than software made for other languages. To improves the model’s accessibility, new data were gathered for additional training during the first stage or for fine-tuning within the second stage. Therefore, we generated a new synthetic Hungarian dataset by developing an existing tool used for another language, TRDGHuMu23, where more than 3M line (text, image) pairs were used for the training phase, and all the methods used to collect and generate these data are shown in Table 1 and fine-tuned with human data. Sometimes, we had trouble reading someone else’s handwriting. It is not only humans who have this problem but also computers. Although computers have been able to recognize and transcribe printed text for decades, recognizing handwritten texts has only been possible for the last few years, particularly for non-English languages. We will investigate the SOTA TrOCR ^1^ vision-language model utilizing transfer-learning technology for the downstream task using A100 NIVDA. The Runtime environment includes A100 NIVDA 8 GPUs with 80 GB RAM Digital Hungarian Heritage Lab (DH-Lab), which is fair because fine-tuning was performed with the same hyperparameters, optimizer, and benchmark dataset.

**Table 1.** Synthetic data for (lines & words) level.

Data	Samples	Language	level
lines-hu-v1	500 000	Hu	Line
lines-hu-v2	500 000	Hu	
lines-hu-v2-1	935 213	Hu	
lines-hu-v3	500 000	Hu	
lines-hu-v4	500 000	Hu	
lines-hu-v5	500 000	Hu	
lines-hu-v7	500 000	Hu	
Brown-lines	96 367	En	
Hu-Words-Dict	60 344	Hu	Word
Hungarian Names	4 478	Hu	
En-Words-Dict	466 479	En	

The digitization of handwritten characters is of paramount importance for the preservation of valuable resources and cultural heritage. For this purpose, OCR systems have been introduced. ^2^ In the case of historical sources, automatic transcription is more difficult owing to a lack of data, increased complexity, and lower quality of resources. To solve these problems, transfer learning or the enhancement of pre-trained language models would be a viable solution. Modern transformer OCR ^3^ pipelines are based on transformer architecture, which consists of a vision encoder and text generator decoder that have been utilized to answer the question: Does pre-training of synthetic data and fine-tuning of human data minimize the error rate?

Building on this foundation, the main objective of this study is to fine-tune such language models by using OCR models pre-trained on an international dataset and then use transformer-based language models for the Hungarian language, such as GPT-2, BERT, PULI _BERT_, RoBERTa, ^4– 7^ with vision models such as Deit ^8^ to enhance the fine-tuning results. Several approaches to enhancements have been explored in this study, the first of which was to use the weights of the pre-trained language model to initialize the decoder in the OCR model. The second approach used the sequence-to-sequence (Seq2Seq) approach to integrate the language and visual models in one OCR architecture. These models were fine-tuned after being pre-trained on letters from János Arany (Provided by ELTE DH-Lab) and evaluated according to the CER and WER metrics. ^9^ The output of this thesis is an extensive study of more than 20 experiments targeting different approaches and detailing the performance increase caused by specific SOTA encoder-decoder pairs and architectural changes. Furthermore, the best-performing model is exported for inference, and a tool that could be used by the faculty of humanities researchers will be developed.

The main contribution can be summarized as the following:

I. We generated around three million (feature, label) pairs for synthetic handwritten data and made them publicly available.
II. Leveraging vision-language pre-trained models in Seq2Seq architecture.
  - Roberta _base_ with a deit
  - PULI _BERT_ with Deit.
III. We see good improvement significantly in the DH-Lab dataset, where the CER and WER were 5.764% and 23.297% has minimized to 3.681% and 16.091%. Thus, the contribution shows the results have been improved by 2.083% and 7.206% for CER and WER, respectively.

So far, there are many reasons that we think leads to this improvement pre-train with more data Syn “e.g.” and data augmentation in an efficient way could lead to more accurate results. All experiments were performed on OCR_HU_Tra2022 repo [1].

The HTR task involves two tasks in the complete life cycle, and this study has limitations, where the focus is on the text generation task. We leave the text detection method for future work models such as YOLOv12, DETER2, ^10, 11^ or any SOTA models could be utilized for object detection or localization. Models are trained and fine-tuned with lines in a complete form file format such as PDF or others, but they need to be prepossessed by line segmentation, which is a limitation here. Additionally, these models are limited to a set of languages.

## Related work

Optical Character Recognition (OCR) is already in an advanced state, whereas Handwritten Text Recognition (HTR) is still in its early phase. ^12^ OCR, an ancient computer vision task, is a popular and ancient technology used to convert images into searchable text, dating back to 1914, and has been used since 2012. Handwritten recognition can be performed online, such as using a whiteboard for handwriting and converting it to text, or offline, as in this case, with scanned images.

**Figure 1.**
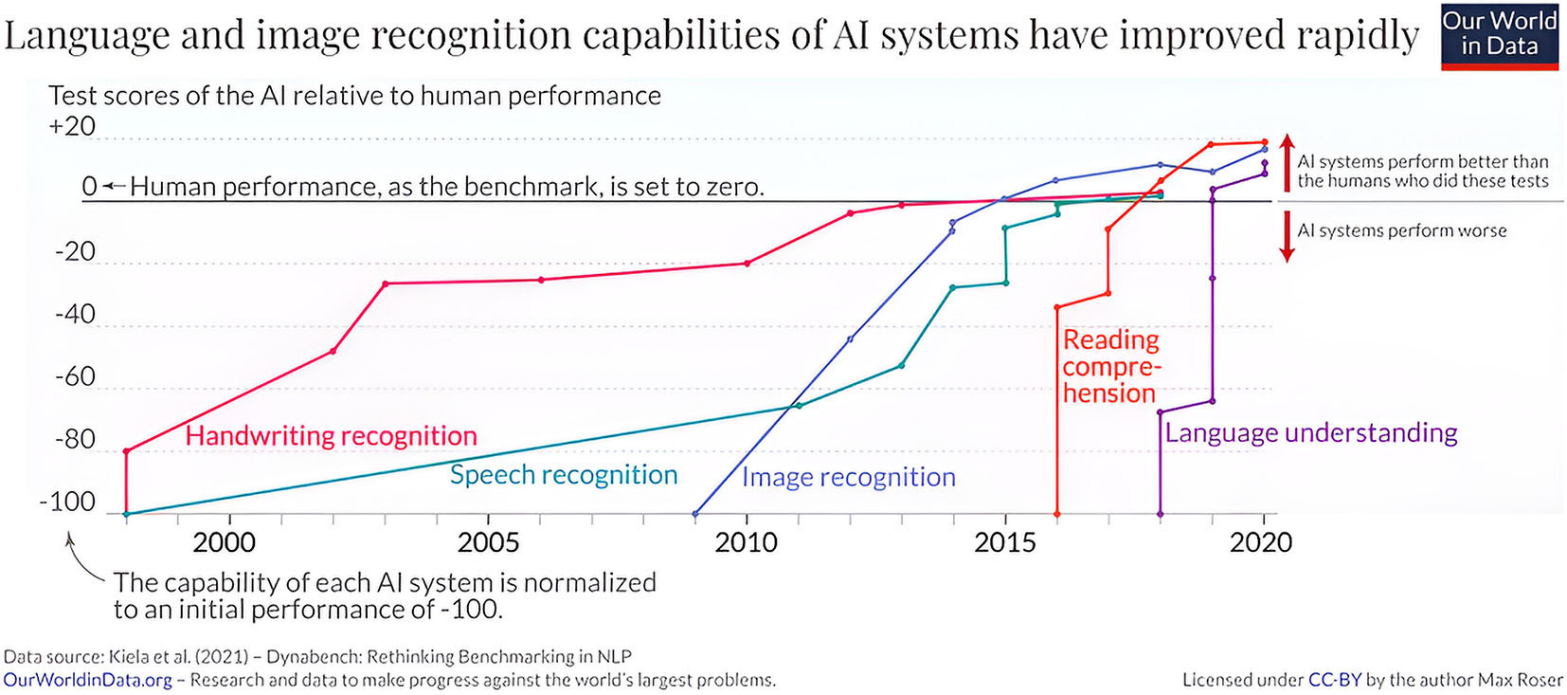
The language and image recognition capabilities of AI systems have improved rapidly. ^8^

Writing recognition uses hardware and software to convert handwritten documents into text for machine reading with transformer-based OCR and pre-trained models such as TrOCR being later developed. OCR technology powers daily systems and services, including document indexing, personal identification, business cards, and automatic number plate recognition. It also helps understand clients and enhance customer service. Kiela et al. (2020) show in Figure 1 the development of handwritten tasks through 21 centuries. ^8^

Tesseract: An open-source OCR engine called Tesseract was created by HP company approximately 40 years ago. Hewlett-Packard built the open-source OCR software Tesseract in the 1980s. Google is responsible for maintaining it. This is a popular solution for numerous OCR workloads because it handles a large number of languages and has been taught in millions of texts. However, it can have difficulties with handwriting and poor-quality scans, and may require significant human adjustment for particular use scenarios. ^13^

Paddle OCR: This is an OCR engine created using open-source DL algorithms. It can handle a variety of documents of different types and accepts many languages, including Chinese and English. It can be tailored to fit particular scenarios and is intended to be simple to use. It is a free tool that can be used for printed text on different platforms such as the web, mobile apps, and Internet of Things (IoT) devices. ^14^

EasyOCR: Another open-source OCR engine with a user-friendly interface. It can handle a variety of document forms covering over 70 languages, including Hungarian. It analyzes papers using DL and, in some situations, can even read handwriting. ^15^

Kera’s OCR is based on Kera’s DL framework. It can handle a large range of document formats and an enormous variety of languages, and can be used for the Hungarian language after fine-tuning. It can be tailored to fit certain use cases and was created to be simple to use. ^16^ This might not be as precise as the many other OCR machines on this list.

Abbyy OCR is a multilingual business OCR technology with an easy-to-use UI that can correctly process complex documents. It might cost more, but it makes use of sophisticated machine learning techniques. ^17^

Google Cloud Vision is a cloud visual solution that uses deep learning methodologies for analyzing files and images, and can detect text in over 50 languages, such as Hungarian. Although free of charge, it is not appropriate for offline operations or information security.

ViTSTR: Similar to ViT is a Visual Transformer, instead of text tokens, it uses parts (patches) of the image as a token sequence and performs classification based on them. ^18^ Simple Transformer Encoder architecture initialized using DeiTs, as they introduced in pre-trained parameters trained beforehand in the MJSynth, SynthText dataset, and augmentations managed to beat the high baseline in accuracy, using the augmentations contributing +(1.5 − 1.8) to accuracy. ^19^

PARSeq: An ensemble of auto-regressive models with a common architecture and parameters can be viewed as performative language modeling trained (PLM-trained), which is a development of auto-regressive modeling. ^20^

Scene Text Recognition (STR): The strategy is to integrate language data indirectly into the STR. The Character module of the tailored adaptive addressing and aggregation module selects a relevant combination of tokens from the ViT of tokens and merges them into a single output token corresponding to that character. ^21^ To implicitly model linguistic information, subword classification heads based on Byte Pair Encoding (BPE) are employed. ^22^

Masked Image Modeling (MIM): Up to 75 percent of the image regions act as masks at the prior-to-training level. The other patches are assigned to the transformer encoder module. The entire set is subsequently processed by the decoder, which reconstructs the pixels of the original image from the encoded representation after adding the appropriate masking signals. MaskOCR is Encoder Representation for the SOTA, where the encoder is ViT, pre-trained self-supervised, and the decoder is DETR, which is a set-based object detector using a transformer on top of a convolutional backbone DETR-style: self-attention. ^23^ In addition to cross-attention, FFN blocks are pre-trained on synthetic text images, while the encoder weights are frozen. ^24^

Masked Vision-Language Transformers (MVLT) are a cutting-edge model structure that integrates written and visual senses to carry out a number of activities, including picture production, visual question answering, image captioning, and HTR. The widely utilized transformer paradigm, which has shown exceptional performance for applications requiring NLP, is extended by MVLT. ^25^ Understanding and creating significant links between visual and textual data are the primary goals of MVLT. To collect long-term reliance and contextual data in both the vision and language domains, it uses the strength of self-attention processes. An additional ensemble approach can be utilized to enhance character token prediction. ^26^

Sequence-to-sequence (Seq2Seq) Modeling: Tasks such as I2T, T2T, ASR, HTR, I2I, and others are considered as Seq2Seq. Seq2Seq architecture allows the model to capture the dependencies and relationships between different elements in the input and output sequences, enabling it to learn complex mappings between sequences of different lengths and structures.

It was first proposed with the use of RNNs ^27^ were used for the first time for sequence classification and then used for ASR, and now we are using it in HTR [2]. TrOCR is an excellent instance of an e2e problem, and there has been a lot of recent work focusing on different pre-training objectives for transformer-based encoder-decoder models, but the model architecture remains largely unchanged. Seq2Seq challenges can be solved using an encoder-decoder design that employs transformers. Transformers were presented for the first time in “Attention is all you need” (Vaswani et al., 2017) ^28^ papers. It uses an attention method to process both input and output patterns.

Connectionist Temporal Classification (CTC): An ANN approach, termed CTC, is utilized to solve Seq2Seq tasks such as speech and handwriting recognition. ^29^ The CTC method is used to teach a network of neurons to transform variable-length input sequences to variable-length output sequences. The output of a sequence can be shorter, longer, or precisely the same length as its input. The CTC algorithm adds a blank symbol to the input sequences, enabling repeated output and variable length models. It solves tasks such as machine translation, HTR, ASR, and Seq2Seq. Although the TrOCR model outperforms this method, it is still a related technique.

The first stressful work related to generating synthetic data used the nearest-neighbor-based collection OCR. ^30^ Writing strategy, in particular, where there are common characteristics in large data, ^31^ is important in developing pattern recognition challenges.

In this study segmentation-based approaches for cursive script recognition (Saba T, 2014), ^32^ On the contrary hand, end-to-end recognition of handwritten content becomes conceivable by current transformer-based techniques.

### Dataset

The dataset used was privately provided by the Hungarian Digital Heritage Lab (DH-Lab). This dataset is the historical handwriting of the famous Hungarian author János Arany. The collection method involves archival research, utilizing private data from DH-Lab and generating synthetic datasets using a public Hungarian corpus to supplement the original handwritten material.

Table 1 shows the data we generated, most of them at the line level and a few at the word level. The data sources are the Hungarian and English Brown corpora [3]. The data used during training is the Hungarian version, with a small English base, where we took samples from this corpus by splitting it into small units and breaking long text into fixed sequences of length between 8 − 12 words per line and cleaning it by keeping only alphanumeric characters with some needed special characters in the first step. In the second step, we reconfigure a new toolkit that generates synthetic data by including Hungarian. All the required development steps can be found on the HuTRDG page of the tool.

*Collected existing datasets*

The TrOCR model’s initial experiments showed poor results because of the need for more data for convergence, prompting the decision to collect publicly available data for fine tuning. STR was collected only for the test dataset, which represents benchmarks collected in one file (CT80-288, ICDAR2013-857, ICDAR-2013-1015, ICDAR-2013-1095, ICDAR-2015-1811, ICDAR-2015-2077, IIIT5K-3000, SVT-647, SVTP-645). ^33^ And We performed our experiment on DH-Lab for Hungarian and IAM/SROIE ^34, 35^ for English to check whether increasing the amount of data minimizes the CER. Table 2 lists the collected data and corresponding number of samples for each.

**Table 2.** Collected datasets where Hu represents Hungarian and En for English.

Data	Samples	Language	Level
DH-Lab (private)	5 995	Hu	Line
IAM ^34^	13 353	En	
SROIE ^35^	52 330	En	
Washingtondb-v1.0 ^36^	656	En	
STR ^33^	11 435	En	Word
washingtondb-v1.0	4 894	En	

*Generate Synthetic (Syn) Hungarian dataset*

**Figure 2.**
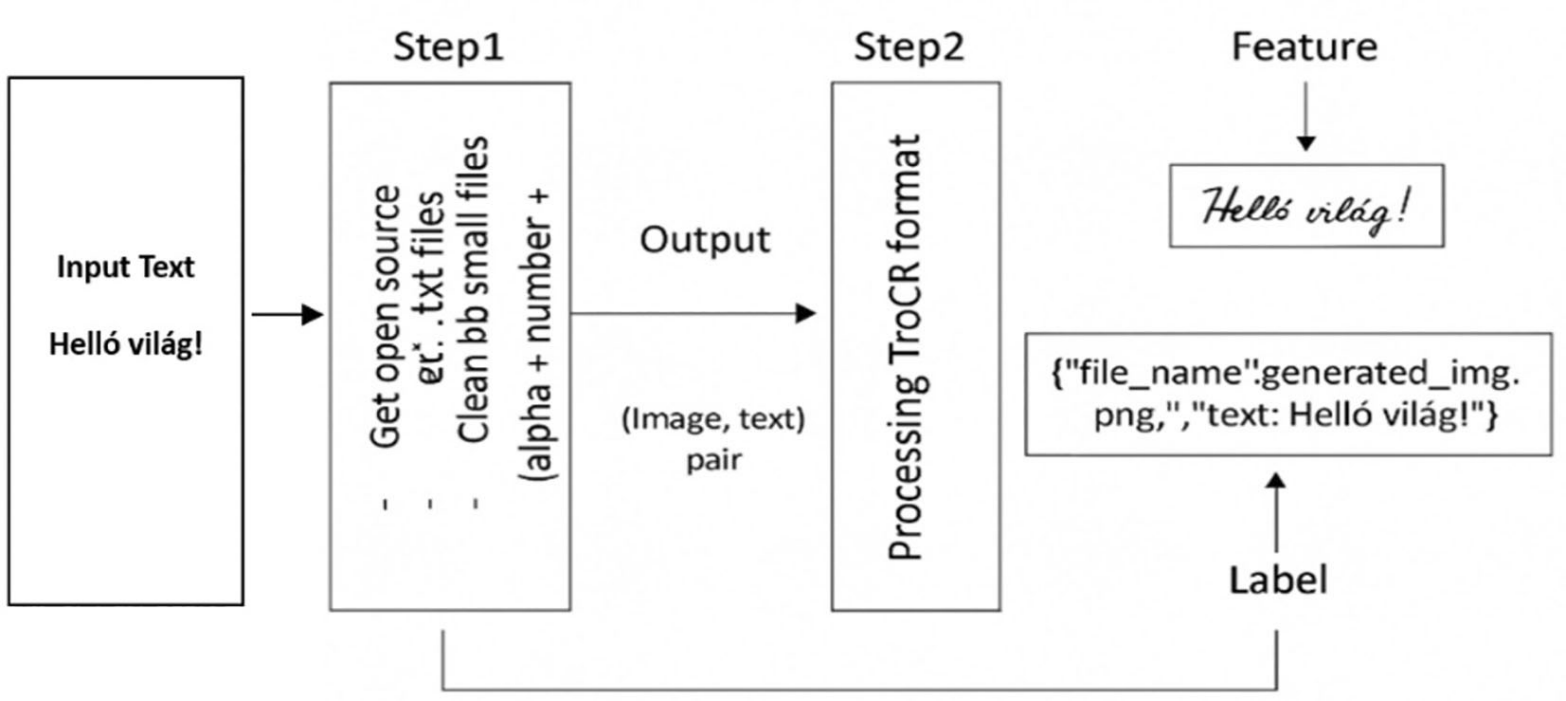
Figure Shows the whole process for Data Generation.

Divergent results were obtained with a limited dataset due to the lack of a Hungarian human-annotated handwritten large dataset. Building on an existing tool for international languages to generate synthetic data. ^37^ We used an open-source font by collecting approximately 200 font types ^38, 39^ based on Hungarian to present synthetic data. We published a 3M set of line-level (image, text) as part of this work by making them publicly available, Data for English and Hungarian was generated data using seven Hungarian versions with varying parameters for augmentation, including blur, Gaussian, distortion, rotation, color, uncolored text, noise, and skewing, with various background images, as shown in Table 1 there are some labeling issues where there are some special Hungarian letters not recognized (á,é,í,ö,ó,ő,ü,ú,ú,ű) for some utilized types of fonts. Figure 2 shows a sample algorithmic procedure for converting plain text into HTR data.

*Data processing and description*

**Figure 3.**
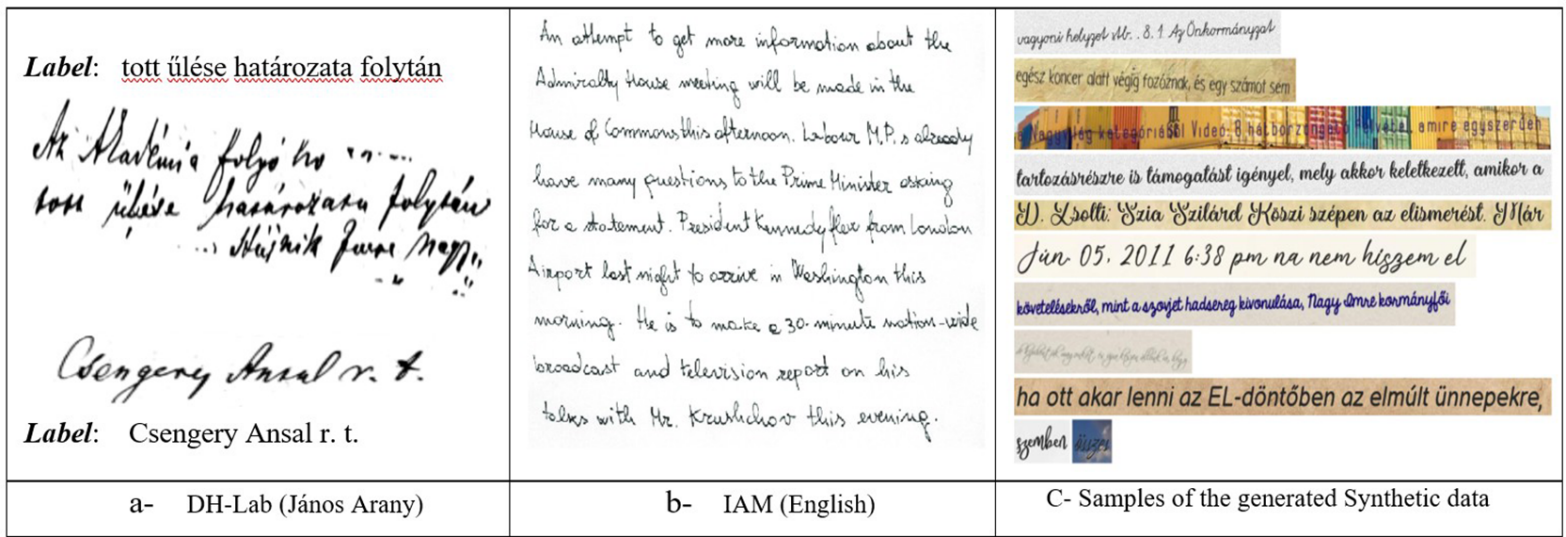
Samples for different datasets used during this study (Hungarian Language).

The JSON line is used to convert images into labels, ensuring efficient data handling for big-data processing. DH-Lab data are very small, human-annotated (around 200 pages) for training, and it is private and contains images (in jpg format). These images were segmented by lines and annotated with the corresponding text in the text file during the text-detection phase. Annotations in the image include the image name, status, and metadata parameters. The text is separated by (|) characters and the (+) sign concatenates the next line with the current sentence. Figure 3 shows that random sampling is used for different datasets, such as the IAM dataset, which has the same raw data format at different levels.

There are three methods for synthetic data generation: programmable algorithmic-based, machine learning (ML), and DL-based, with the latter recommended for more human-like handwritten data, but fine-tuning or pre-training on Hungarian text. ^40^

*Data Augmentation (Aug.) in an efficient way*

**Figure 4.**
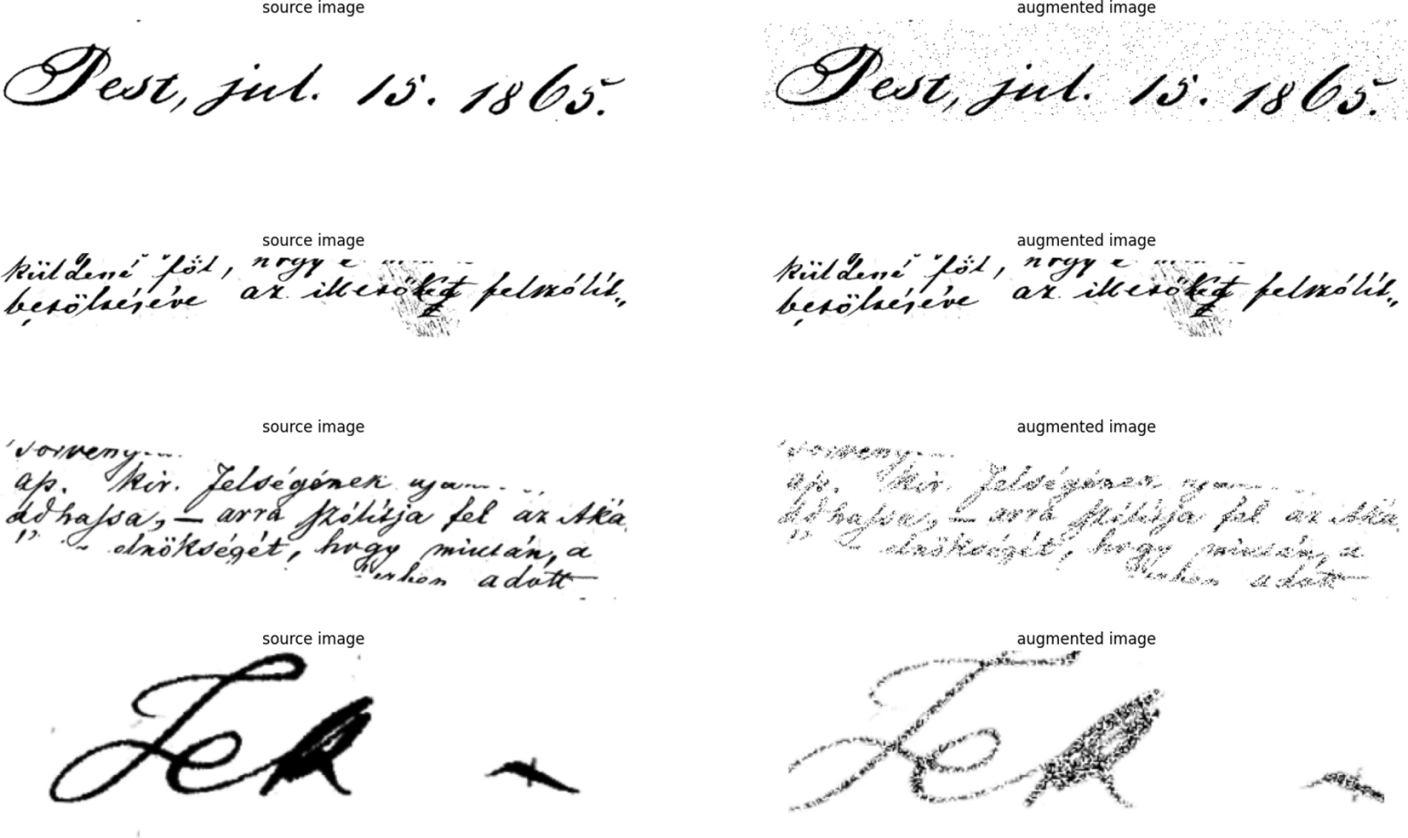
The figure shows different augmentation methods; the left-side images are the source, and the right side is the resulting augmented image.

We enhanced the DH-Lab dataset using augmentation and CV methods, thereby reducing overfitting and improving generalization. The DH-Lab data are grayscale, focusing on grayscale areas. Morphological alterations alter the appearance of text lines via expansion and erosion. Noise introduction involves pixel color insertion or elimination using dark colors with random distribution for recognition difficulty. Sporadic showers add a rain-like appearance to the image. This approach has been successful with TrOCR and should function effectively in any additional system. ^41^ Three sets were generated from a single dataset. 10% of test sets, 10% of validation, and 80% of train sets. Figure 4 shows random samples from the Aug. data.

## Methodology

**Figure 5.**
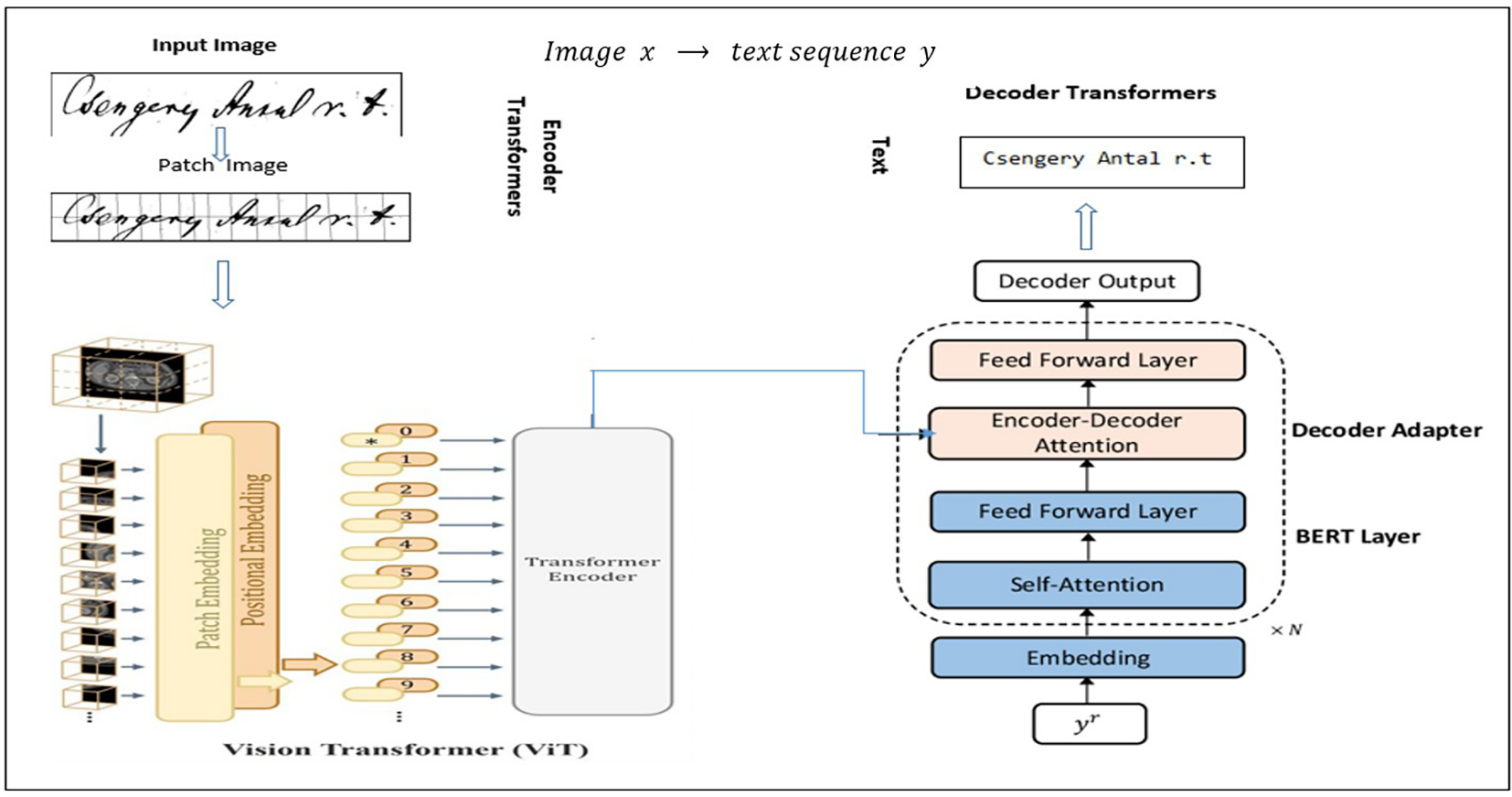
Leveraging vision-language (ViT ^18^ + Bert ^5^) models in Seq2Seq architecture.

Sharing the parameters with the intended learners immediately is a straightforward technique for controlling these parameters. An image encoding module based on ViT and a sequence generation decoder based on transformers improved by language model features are assembled in Figure 5 to create a vision-to-text workflow for Hungarian handwritten text recognition. The handwritten text picture provided was first separated into patches in this process, and these patches were then embedded and enhanced with spatial data. The Vision Transformer encoder layers evaluate these embeddings, enabling the computational model to extract contextual and spatial characteristics using a script. The encoded visual representation is then fed into a transformer decoder, which uses encoder–decoder attention to associate the visual attributes with the produced text and self-attention layers to analyze previous output tokens to perform sequence modeling. Furthermore, BERT layers were added to the decoder to improve contextual knowledge, especially for materials in Hungarian. Text that had been broken down into tokens (words or subwords) was processed by BERT. Embeddings were generated using tokens. Self-attention is used through a transformer encoder for interpretation. BERT can comprehend word relationships and meanings by utilizing contextualized visualizations of the text. Ultimately, the decoder transforms the handwritten source into its digital format by generating an identified string. TrOCR is basically a ViT ^18^ Encoder + Transformer Decoder trained for sequence-to-sequence (Seq2Seq) mapping:

Input image processing divides the input image I $\in R^{H\;x\;W\;x\;C}$ into N patches and embeds each patch as the embedding vector in Eq. (1):

$$x_{0}=\left[E\left(p1\right);E\left(p2\right);\ldots ;E\left(\mathrm{pN}\right)\right]+P$$ (1)

Where E (p _i_) is the linear embedding of patch p _i_ and P is the positional encoding in Eq. (1) and Eq. (2)

The vision transformer encoder processes the patch embeddings through *L* transformer encoder layers:

$$z_{L}=\mathrm{Vi}T_{\text{Encoder}\left(x_{0}\right)}=\text{EncoderLayerL}\left(\ldots \text{EncoderLayer}1\left(x_{0}\right)\ldots \right)$$ (2)

The next representation will be the transformer decoder with the language model. At each decoding step ***t***, predict the next token ***y***_***t***_ given previous tokens **y**_**< t**_ and encoded features **z**_***L***_ in Eq. (3), the decoder starts with a special character *[s]* start of the token and ends with *[/s]* end of the token.

$$y_{t}={\text{DecoderLayer}}^{M}\;\left(y<t,z_{L}\right)$$ (3)

The decoder includes a self-attention mechanism to capture the dependencies in the previous tokens. Encoder–decoder attention aligns the visual features z _L_ with output tokens. Language model integration, that is, (BERT), enhances contextual understanding, as shown in Eq. (4).

$${\tilde{y}}_{t}=\text{BERT}\left(y_{t}\right)$$ (4)

The next Eq. (5), and Eq. (6), represent the output text generation for the final predicted token sequence in short is:

$$\hat{Y}=\left[{\tilde{y}}_{1},{\tilde{y}}_{2},{\tilde{y}}_{3}\cdots {\tilde{y}}_{T}\right]$$ (5)

$$\hat{Y}=\text{Decoder}\left(\text{BERT}\left(\text{Decoder}\left(y<t,\mathrm{Vi}T_{\text{Encoder}\left(x_{0}\right)}\right)\right)\right)$$ (6)

*Encoder*

**Figure 6.**
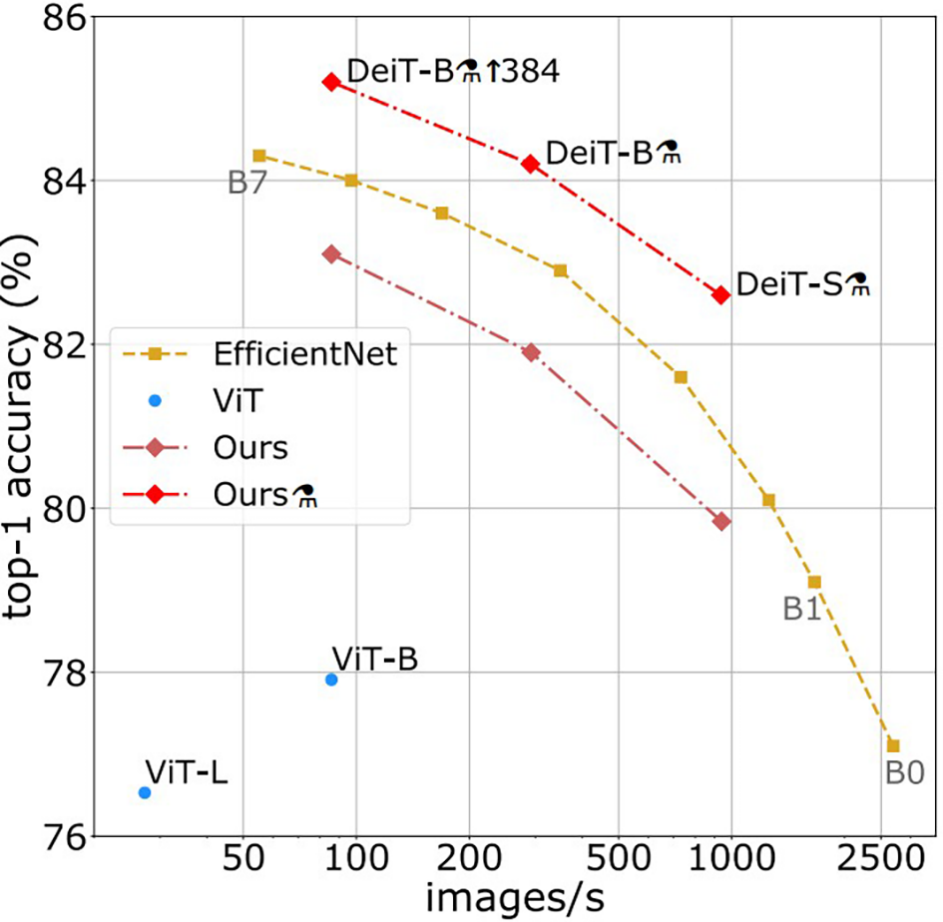
Throughput and accuracy on ImageNet. ^3^

The Encoder here represents the visual part in the TrOCR architecture, and it is introduced in Figure 5, where the image is broken into a series of 16 × 16 patches, which are utilized as the text to be entered into the image, after first resizing the text being the (normalized) input image to 384 × 384. Some of models based 224∗224 “e.g.” ViT model to extract the features and encode them we use a list of possible vision transformers models for image understanding: vision encoders (like ViT represent one of the SOTA in CV and are widely employed for various image identification applications, and have a strong competitor in the shape of Vision Transformer (ViT)). In terms of computational effectiveness and accuracy, the ViT models perform nearly four times better than the most advanced CNNs currently available (similar to the BERT model). The model is shown in the images above as a series of fixed-size patches. One randomly masks off a significant number (75%) of the picture patches during pre-training. The visual patches were first encoded with the encoder, and the positions of the masked patches were then inserted with learnable (shared) mask tokens. The decoder reconstructs the raw pixel values for masked locations using encoded visual patches and mask tokens as inputs. Distilled Data-efficient Image Transformer (base-sized model): pre-trained model on ImageNet-1k (1 million pictures, 1,000 classes) at resolution 224 × 224 and fine-tuned at resolution 384 × 384 DeiT. As shown in Figure 6, it was introduced in the training of data-efficient image transformers and distillation through attention. It is a transformer-specific teaching technique for students. It depends on a distillation token to ensure that the pupil pays attention to and learns from the teacher. Moreover, it outperforms the results achieved by the ViT model. During the experiment, excellent results were obtained by leveraging the PULI _BERT_ and Roberta _base_ models. Let us take, that is, the Vision Transformer (ViT) starts with Patch Embedding by splitting the input image x $\in R^{H\;x\;W\;x\;C}$ into patches of size *P×P*, flattening, and projecting to dimension d, Eq. (7).

$$z_{0}=\left[x_{P}^{1}\;E_{,}\;x_{P}^{2}E;\ldots ;x_{P}^{N}\right]+E_{\mathrm{pos}}\;\text{where}\;N=\frac{\mathrm{HW}}{P^{2}}$$ (7)

***E*** is the learnable matrix of patch embedding and positional embedding is ***E***_***pos***_. Transformer encoder layers.

Transformer encoder layer includes a multi-head self-attention (MSA) block followed by a feed-forward network with residual connections and layer *l* normalization from 1 to *L*, as shown in Eq. (8) and Eq. (9):

$$z_{l}^{\prime }=\mathrm{MSA}\left(\mathrm{LN}\left(z_{l}-1\right)\right)+z_{l}-1$$ (8)

$$z_{l}=\mathrm{MSA}\left(\mathrm{LN}\left(z_{l}^{\prime }\right)\right)+z_{l}^{\prime }\;$$ (9)

ViT maintains pictures that can be classified into micro-areas. When flattening, each individual patch is converted into a vector. The result of this process is a series of vectors that depict the visual elements of the graphics. Think of ViT as “reading an image the same way a Transformer reads words.”

*MSA* is the multi-head self-attention, *LN* is the layer norm, *MLP* is a position-wise feed-forward network, and the output of the encoder is as follows: *h _enc_ = z _L_*

**Figure 7.**
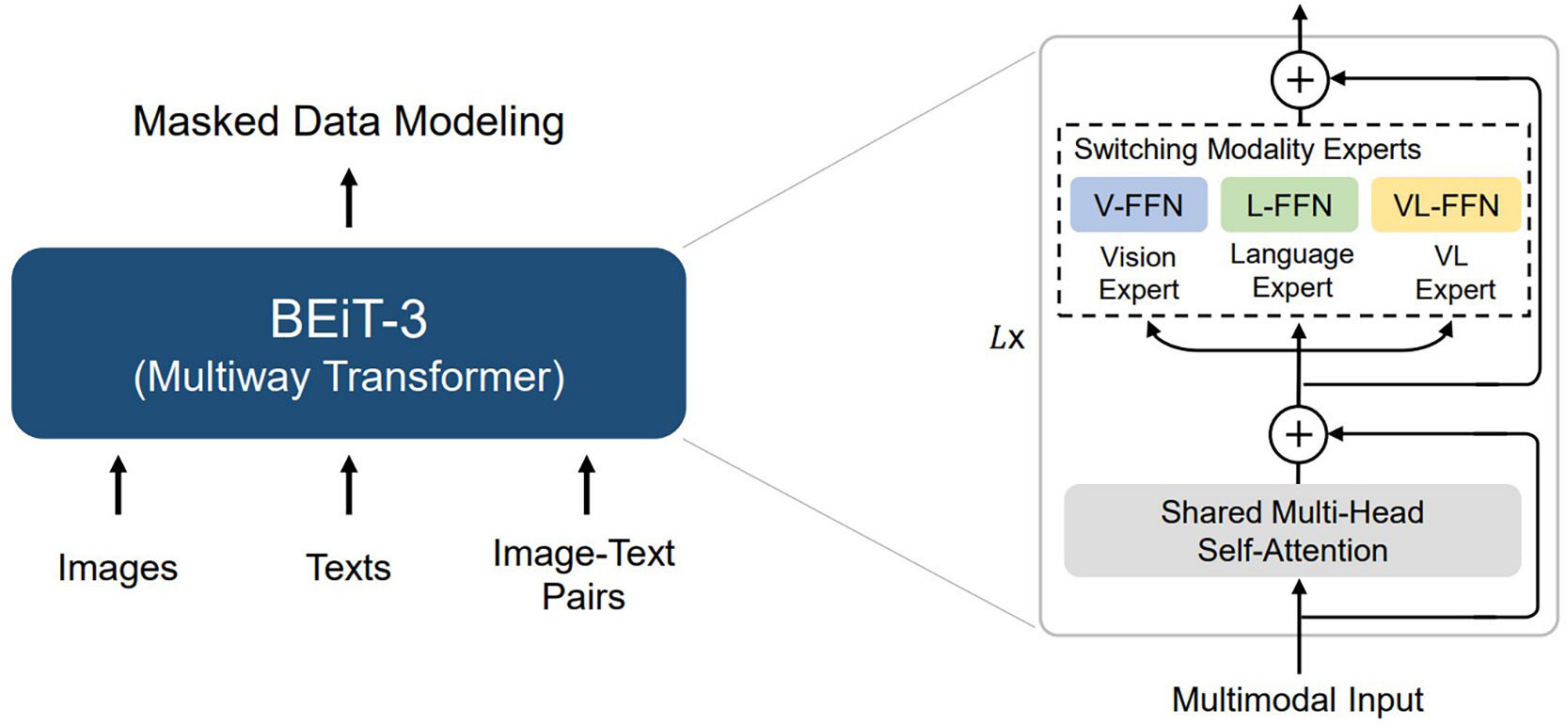
Encoder example (BEiT-3). ^42^

Figure 7 shows that there is a new release that achieves SOTA for the BEiT-3 model, which we leave for future work. This transformer model is used for tasks such as I2T and VQAv2, which deal with The Microsoft research group BEiT-3, which introduced Vision as a Foreign Language and is BEiT preparing for every vision and image-language activity. (BERT Pre-training of Image Transformers), ^42^ a general-purpose SOTA multimodal basis approach to problems involving visual perception and language that advances significant network structure convergence, pre-training tasks, and model scaling. In addition to BEiT, ^35^ Swin ^43^ was used, but this was the focus of this research. Several visual models can be used, such as ViT, BeiT, Swin, and DeiT. Additionally with only minor differences in CER and WER, evaluated several alternative visual encoders Transformer, Swin Transformer, DeiT, and BEiT within the same TrOCR Architecture. The outcomes showed comparable performance across the tested models, for future work direction the ConvNeXt model can be explored as additional architectures.

*Decoder*

We used different types of text generation models that are based on transformer architecture, such as huBERT, ^44^ Bidirectional Encoder Representations from Transformers (BERT), Distilbert, ^45^ mGPT, ^46^ Generative Pre-trained Transformer (GPT-2), and Bart. ^47^ Where the Bert family was used to living.

The Robustly Optimized BERT Approach (RoBERTa) is a self-supervised transformer model pre-trained on a large corpus of English data, specifically designed for Masked Language Modeling (MLM). It randomly selects 15% of input words to be hidden, contrast to conventional RNNs and autoregressive models like GPT.

PULI _BERT-large_ is a Hungarian Megatron BERT model based on Megatron-DeepSpeed training. The best checkpoint was 1500 K steps, and the dataset utilized was 36.3 billion words. ^48^ The transformer decoder includes the input embeddings. Text tokens ***y***_***< t***_ (previous outputs) are embedded Eq. (10), followed by Masked Self-Attention to prevent looking at future tokens, as shown in Eq. (10), and Eq. (11).

$$e_{t}=W_{e}y<t+E_{P}^{d}$$ (10)

$$u_{l}^{\prime }={\mathrm{MSA}}_{\text{mask}}\left(\mathrm{LN}\left(u_{l}-1\right)\right)+u_{l}-1$$ (11)

The following representation of Eq. (12), is the cross-attention with encoder output, and the decoder attends to the encoder’s visual features:

$$u_{l}^{\prime \prime }=\mathrm{MCA}\left(\mathrm{LN}\left(u_{l}^{\prime }\right),h_{\mathrm{enc}}\right)+u_{l}^{\prime }$$ (12)

The final probability distribution for the next token, shown in Eq. (14) after the feedforward Eq. (13)

$$u_{l}=\mathrm{MLP}\left(\mathrm{LN}\left(u_{l}^{\prime \prime }\right)\right)+u_{l}^{\prime \prime }$$ (13)

$$P\left(y_{t}|y<t,x\right)=\text{Softmax}\left(W\;\circ \;u_{L}^{t}\right)$$ (14)

The loss function utilized was the cross-entropy loss for all tokens in Eq. (15)

$$L=-\sum_{t=1}^{T}\log\;P\left(y_{t}|y<t,x\right)$$ (15)

*Text generation*

**Figure 8.**
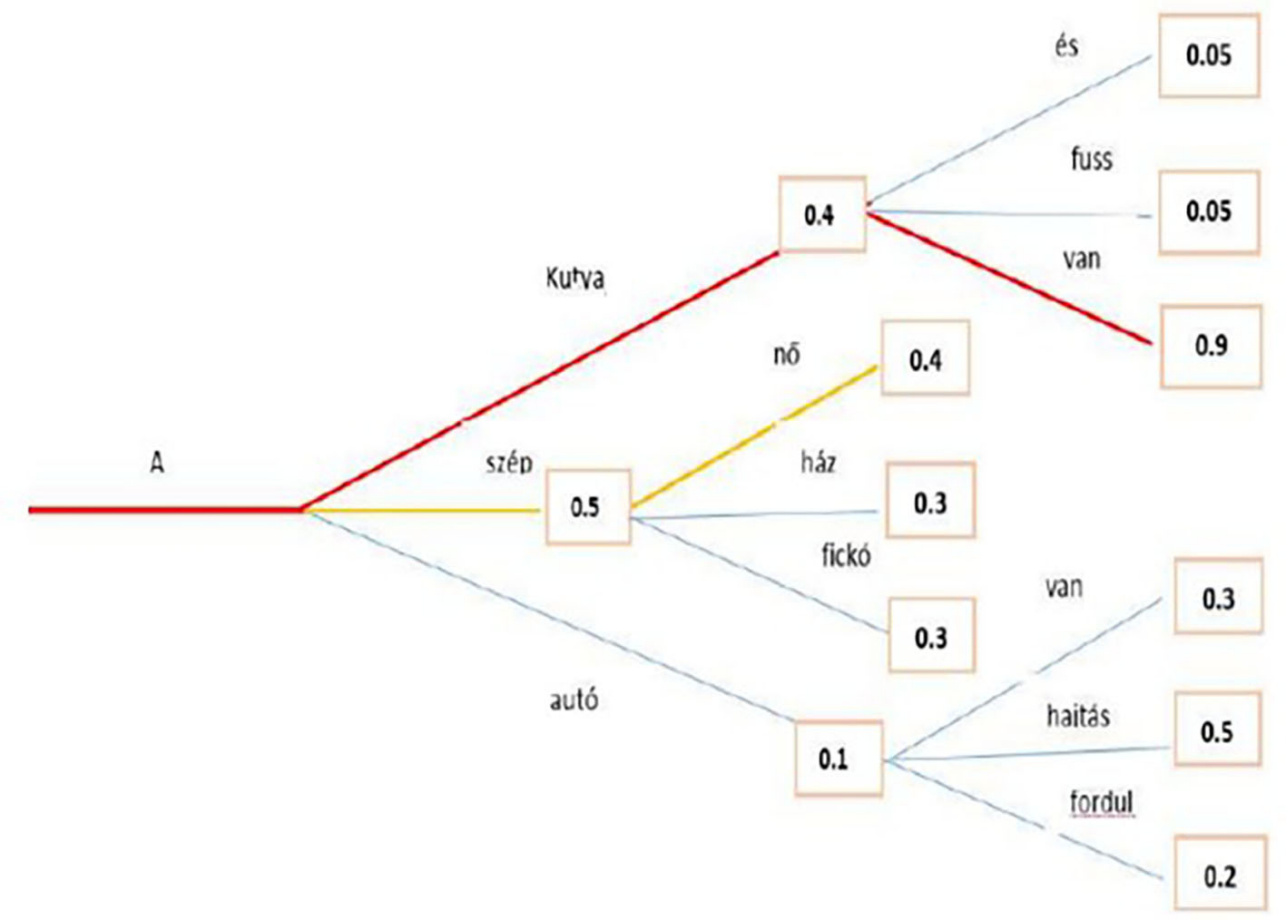
Beam Search algorithm with the Highest Probability.

The beam search minimizes the chance of overlooking hidden high-probability word combinations by maintaining the most likely number of beams at every step and selecting the possibility with the greatest overall likelihood. In a case study, a beam search identified the most probable word sequence in the Hungarian language model. Both greedy and beam searches have close to ***0*** probability to produce the best sequence for long sequences, but beam search converges to a more optimal one, as shown in the example in Figure 8, where the red line represents the path for beam search.

**Figure 9.**
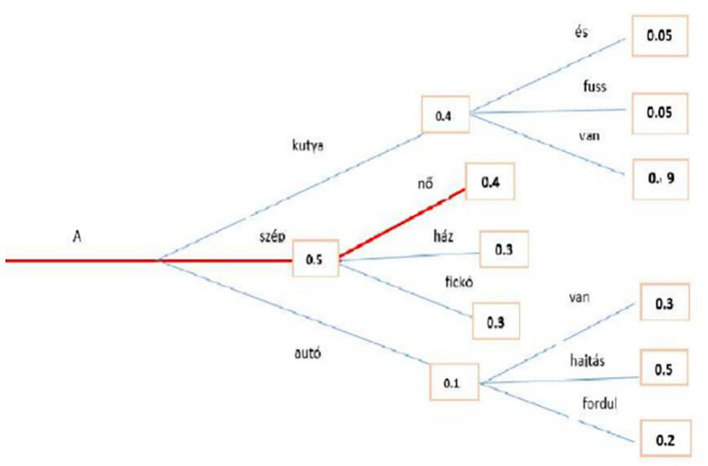
An example for Greedy Search algorithm with the Highest Probability.

The second methodology used to generate text is Greedy Search: at each time step *t*, greedy search only selects the next word *w* with the highest probability *P*, and the conditional probability is shown in Eq. (16). While the Figure 9 above shows an example that starting from the word “A” the algorithm greedily chooses the next word of highest probability, “szép” and so on so that the final generated word sequence is (“A,” “szép,” “nő”) having an overall probability of 0.5∗0.4 = 0.20. This method showed an unsuccessful search, which is highlighted by the red line. Transformers can use a greedy search. However, the model begins to cycle. This is a fairly common challenge for text generation in language models, and it seems to be particularly common in greedy and beam search. ^49^

$$w_{t}=\mathrm{av}g_{w}\max P\left(w|w_{1:t-1}\right)$$ (16)

$$P\left(w_{1},w_{2},\ldots ,w_{T}\right)=\prod_{t=1}^{T}P\left(w|w_{1:t-1}\right)$$

Where:

• w *_t_* is the word chosen at time step *t and* w *_1:t−1_* is the sequence of previously generated words.
• P(w|w *_1:t−1_*) represent conditional probability of word www given the previous words.

*Evaluation metrics*

Character and Word Error Rate are metrics used to evaluate Automatic Speech Recognition (ASR) techniques, similar to HTR tasks, and seq2seq modeling requires sequence-level evaluation. ^50^ The Word Error Rate (WER) is a crucial indicator of an HTR system’s performance; however, its accuracy is limited because of the potential for a different word sequence from the reference. The WER is derived from the Levenshtein distance, but further research is needed to understand the exact nature of the HTR problems. The WER was calculated using Eq. (17),

$$\mathrm{WER}=\frac{S+D+I}{N}=\frac{S+D+I}{S+D+C}$$ (17)

Where *S* is the number of substitutions, *D* the number of deletions, *I* the number of insertions, *C* the number of correct words, and *N* the number of words in the reference ( *N = S+D+C*). Word accuracy: W _Acc_ = 1-WER.

The Character Error Rate (CER) is a commonly employed measure of how well an automatic speech recognition system performs. CER acts on characters rather than words, analogous to word error rate (WER). The character error rate is calculated using Eq. (18):

$$\mathrm{CER}=\frac{S+D+I}{N}=\frac{S+D+I}{S+D+C}$$ (18)

where *S* is the number of substitutions, *D* the number of deletions, *I* the number of insertions, *C* the number of correct characters, and *N* the number of characters in the ground truth ( *N = S + D + C*). Character accuracy: *C _Acc_ = 1 – CER.*

*Settings*

We set several beams greater than one and used a 4. It is advised to use up to 10 as TrOCR researchers have utilized it. In addition to early stopping to reduce carbon emotion and save resources, the number of repeated n-grams = 2 so that no 2−gram appears twice. The optimizer is AdamW, where Adam’s betas parameters (b1, b2), weight _decay_ = 0 and beta1 = (0.9, 0.999), and the initial learning rate (LR) is maintained at 2e – 5.

Detailed error analysis by character type (diacritics and ligatures) may be consider as limitation in this study. Word occurrences exceed the stated Character Error Rate as well as Word Error Rate.

## Experiments

This study explores the improvement of SOTA for HTR approaches in Hungarian, presenting line-level test results and word-level experiments. The methodology involves experiments for model selection, incorporating English and Hungarian databases and utilizing leveraged models such as Roberta _base_ and PULI _BERT_ with Deit, including synthetic data experiments. The results were evaluated based on the CER and WER metrics. Permutation of the model selection experiments was performed, and three models were selected: TrOCR _large_ handwritten, Roberta _base_, and PULI _BERT_ with Deit. TrOCR _large_ was chosen for synthetic (Syn) Hungarian pre-training (Stage-1), followed by the TrOCR base encoder with the best Hungarian and international text models. Experiments were evaluated using DH-Lab data at the second (Stage-2). Experimental results show that TrOCR _large-handwriting_ is the best for training on the same domain data pattern, indicating that generating Syn Handwriting data can enhance the accuracy of the results without using our methodology.

## Results and discussion

**Figure 10.**
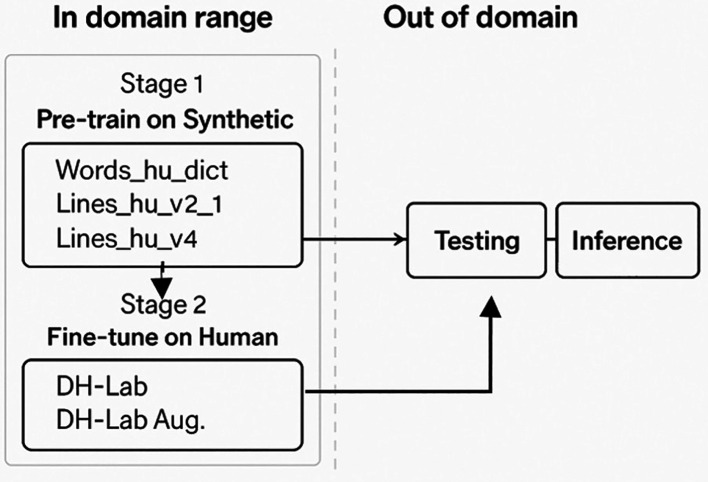
The figure shows the procedure for the methodology used.

Figure 10 shows that the proposed methodology has two stages: the first is the pretraining for TrOCR models or the new leveraged models in the Seq2Seq architecture with Syn data, and the second stage is to fine-tune the pre-trained models on human data (DH-Lab). We show the Val _**Cer,**_ Val _**Wer**_, Test _Cer **,**_ and Test _Wer_ metrics for the proposed experiments.

### Task: DH-Lab

Table 3 shows the baseline model, the TrOCR _large, printed_, and the fine-tuned results show that the best Val _Cer_ 4.447 in TrOCR _large_ and the best Val _Wer_ is 19.806 for Hungarian language and 0.1003, 2.571 CER, and WER for IAM (English) data, respectively. We will see further improvements when dealing with the Syn method.

**Table 3.** Testing baseline models results for fine-tuned (Hu Lines Level) on validation set.

Model id	Data	Steps(K)	Aug.	Val _Cer_	Val _Wer_
TrOCR _large handwritten_	DH-Lab	8	×	5.764	23.297
	DH-Lab	8	×	**4.447**	**19.806**
	IAM (En)	8	×	0.1003	2.571
	DH-Lab	8	✓	5.221	22.211
TrOCR _large printed_	DH-Lab	8	×	6.0731	24.603
	DH-Lab	8	✓	6.473	22.211

### Task: SROIE

The second experiment was the Scanned Receipts in English Language (SROIE) dataset, which is based English language, the lower CER is 1.421 and the WER is 6.852 obtained on the test set for the TrOCR _base_ model. Table 4 shows the rest of the other models, such as Bert _base-uncased_, Hu Bert, PULT _Bert_, and Roberta _base_, show acceptable, reasonable error rates and could be improved by using the proposed two-stage methodology, in particular, Roberta _base_ + Deit and PULT _Bert_ + Deit, and we choose them besides the TrOCR in the next experiments.

**Table 4.** Testing Results on SROIE (Task: SROIE) on line level except the last two rows in sentence level.

Data	Steps(K)	Data	Train _Loss_	Val _Loss_	Test _Cer_	Test _Wer_
TrOCR _base_	24	SROIE	0.011	0.129	**1.421**	**6.852**
Roberta _base_ + Deit	34		0.0217	0.595	7.996	20.217
PULT _Bert_ + Deit	4		0.4964	0.532	16.358	21.133
Roberta _base_ + Deit	90	SROIE+IAM	0.0002	0.514	9.996	14.586
Vit + hu _Bert_	20	SROIE	1.885	4.315	54.028	88.511
Vit +Bert _base-uncased_	7		0.1431	3.119	61.394	75.572

TrOCR variants, showing that TrOCR _small_ (62M parameters) is the quickest, processor 8.37 sentences per second, whereas TrOCR _base_ (334M) and TrOCR _large_ (558M) are slower but more sophisticated. Wider models may increase accuracy, but this falls at the expense of significantly reduced execution velocity for the rest of the architecture. Table 5 shows that TrOCR _small_ is the fastest Hungarian handwritten text recognition procedure (8.37 sentences/s) and is best suited for immediate use. The TrOCR _base_ and TrOCR _large_, albeit less rapid, may offer greater reliability, which is beneficial when dealing with a variety of handwriting styles and Hungarian diacritical marks. During testing, the TrOCR _large_ model (16K steps) produced a test CER, WER of 0.7642%, 23.297%, a validation CER of 6.617%, a validation WER of 24.485%, and a training loss of 0.0077. With a substantially greater validation CER of 1.1107%, validation WER of 3.0673%, and test CER, WER of 6.473%, 22.211%, training loss decreased to 0.0013 while augmented (TrOCR _large Aug_), demonstrating that augmentation greatly improved generalization and minimized recognition errors.

**Table 5.** Fine-tuning all the baseline TrOCR models handwritten versions on DH-Lab.

Data	Steps(K)	Train _Loss_	Val _Loss_	Val _Cer_	Val _Wer_	Test _Cer_	Test _Wer_
TrOCR _large_	16	0.0077	0.611	6.617	24.485	**5.7642**	23.297
TrOCR _large Aug_	16	**0.0013**	**0.0615**	**1.1107**	**3.0673**	6.473	**22.211**
TrOCR _base_	4	0.0009	0.559	6.655	25.122	×	×
TrOCR _small_	2	0.8095	0.8463	10.345	37.463	×	×
TrOCR _base-large_	16	0.0077	0.611	6.617	24.485	×	×
TrOCR _base-small_	7	3.3598	3.488	79.41	94.975	×	×
TrOCR _base-small-stage1_	10	2.8583	3.414	79.676	94.009	×	×
TrOCR _base-stage1_	10	0.1017	1.768	24.584	61.554	×	×
TrOCR _stage1_	8	0.0048	1.1614	6.4037	23.6714	×	×
TrOCR _base-large-stage1_	10	0.0062	2.6111	19.815	57.489	×	×
TrOCR _large-stage1_	3.50	0.8025	0.663	9.739	35.362	×	×
TrOCR _small-stage1_	10	0.0062	2.6111	19.815	57.489	×	×

**Figure 11.**
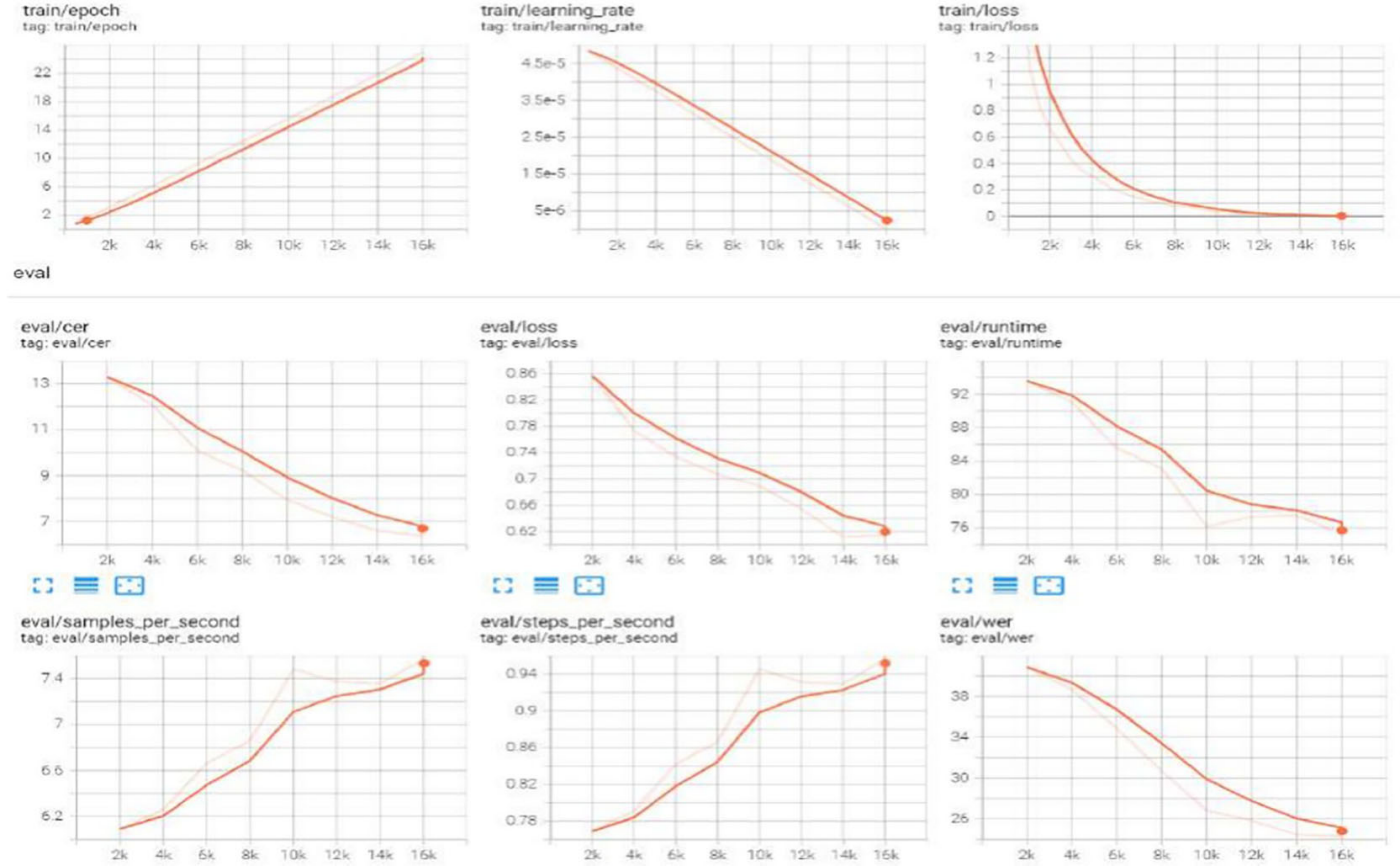
Logs for TrOCR large model on DH-Lab data.

Log visualization aids in understanding the training and evaluation processes, spotting problems, and tracking performance. The Hugging Face (HF) library is a popular open-source tool for NLP jobs that offers various tools and utilities for various models, including log visualization. Figure 11 shows the logits in the tensor board visualization tool for the best obtained results in Table 4 (TrOCR). The CER and WER curves decreased owing to the adaptation of the LR scheduler to logs such as training loss, validation metrics, and runtime data.

### Task: Synthetic hungarian words level

We saw statistics about the collected and generated samples in Table 1, word-level for both English and Hungarian. Table 6 shows that the TrOCR _based_ scenarios, especially TrOCR _large_ (25K steps), provide an ideal balance between test accuracy and validation in Stage-1 testing on Hungarian synthetic word-level data, with test CER, WER of 2.678%, 10.043%. Smaller versions, such as the TrOCR _base_ and TrOCR _tiny_, were successful and performed fairly well. Although Roberta _base_ + DeiT surprisingly achieved the least known test CER (1.875%) and WER (7.684%), hybrid models like Roberta + DeiT and PULI _Bert_ + DeiT, displayed greater validation errors. Overall, the results support TrOCR frameworks as the best option for handwritten text recognition at the synthetic level in Hungarian.

**Table 6.** Testing results from words Hungarian Syn level words-hu-dict (Stage-1).

Data	Steps(K)	Train _Loss_	Val _Loss_	Val _Cer_	Val _Wer_	Test _Cer_	Test _Wer_
TrOCR _large_	25	0.018	0.306	2.842	11.314	2.678	10.043
TrOCR _large_	160	0.0264	0.378	2.906	11.113	×	×
TrOCR _base_	55	0.0055	0.2658	**2.6955**	10.5568	×	×
TrOCR _small_	40	0.0126	0.298	2.729	**9.909**	×	×
TrOCR _base-large_	140	0.0264	0.3783	2.9063	11.113	×	×
Roberta _large_ + Deit	50	1.7992	12.612	6.26	17.104	×	×
Roberta _base_+ Deit	50	0.2076	0.6417	7.6855	30.879	**1.875**	**7.684**
PULI _Bert_+ Deit	50	0.0036	0.765	7.1440	23.674	7.2765	23.5

In this experiment, the TrOCR large training took over four days with a single GPU, whereas PULI _BERT_ with Diet took 14 h and Roberta _base_ with Deit 11 h.

### Task: Pre-train on Synthetics Hungarian hu-lines-v2-1(Stage-1) and Fine-tuning on DH-Lab (Stage-2)

In the next and last experiments, we can see the pre-training (stage-1), Table 7 on synthetic data, and fine-tuning (stage-2) on human data. TrOCR _large_ produced the best overall performance in Stage-1 pre-training on the hu-lines-v2-1 synthetic Hungarian dataset, showing a low validation CER and WER of 1.737%, 4.786% and corresponding test accuracies (1.792%, 4.944%). Competitive results were achieved by Roberta _base_ + DeiT and PULI _Bert_ + DeiT, with PULI significantly surpassing Roberta on test WER. TrOCR base demonstrated worse efficacy in this configuration, with greater validation errors (3.213% and 11.045%) and no test outcomes based on this data. Pre-training (Stage-1) on a single GPU, epochs set to 25, sequence length to 128 in TrOCR _large-handwritten_, 96 for both PULI _Bert_ and Roberta, the LR is 5e -5 and the batch size is 24 for Roberta is 32 the TrOCR _large-handwritten_, and the batch size is set to 100. The TrOCR _large-handwritten_ took more than two months to complete, while Roberta _base_ and PULI _Bert_ took more than three weeks.

**Table 7.** Pre-training on Synthetics Hungarian hu-lines-v2-1 dataset (Stage-1).

Data	Steps(K)	Train _Loss_	Val _Loss_	Val _Cer_	Val _Wer_	Test _Cer_	Test _Wer_
TrOCR _large_	85	0.0259	0.073	1.737	4.786	**1.792**	4.944
TrOCR _base_	15	0.1995	0.171	3.213	11.045	×	×
Roberta _base_+ Deit	260	0.0426	0.0979	**2.264**	6.1205	2.327	6.2332
PULI _Bert_+ Deit	200	0.0558	0.142	2.416	**5.629**	2.129	**4.691**

After Stage-1 pre-training using hu-lines-v2-1 synthetic Hungarian data, the Stage-2 fine-tuning results on the DH-Lab benchmark are shown in Table 8. TrOCR _large_ obtained good results before augmentation (Test CER, WER = 3.681%, 16.189%), but during augmentation, its validation CER fell precipitously to 1.087 percent, even though the test CER increased to 5.221 percent, indicating potential overfitting of supplemented data. With augmentation, Roberta _base_ + DeiT significantly improved, reducing the test CER from 8.374 to 4.889 percent and the validation CER from 9.253 to 2.598 percent. Augmentation additionally supported PULI _Bert_ + DeiT, reducing the validation CER from 7.655% to 1.504%, while test CER improved slightly (5.381% → 6.123%). Overall, Table 7 shows that while augmentation greatly enhances the validation performance for all of them, its influence on the test accuracy differs depending on the model’s structure. Our results exceed the existing literature.

**Table 8.** Fine-tuning Synthetics Hungarian hu-lines-v2-1 on DH-Lab Benchmark dataset (Stage-2).

Data	Aug.	Steps(K)	Train _Loss_	Val _Loss_	Val _Cer_	Val _Wer_	Test _Cer_	Test _Wer_
TrOCR (Palkó G, 2023) ^51^	×	×	×	×	×	×	5.86%	×
Transcribus (Palkó G, 2023) ^51^	×	×	×	×	×	×	9.30%	×
TrOCR _large_ ( **our**)	×	16	0.0022	0.449	4.343	17.931	**3.681**	**16.189**
	✓	163	0.0002	0.061	**1.087**	**2.527**	5.221	18.46
Roberta _base_+ Deit ( **our**)	×	1	0.0464	0.6248	9.253	29.9507	8.374	29.121
	✓	12	0.0008	0.106	2.598	6.218	4.889	18.558
PULI _Bert_+ Deit ( **our**)	×	2	0.0088	0.691	7.655	22.557	5.381	**16.091**
	✓	26	**0.0**	0.072	1.504	2.982	6.123	16.357

**Figure 12.**
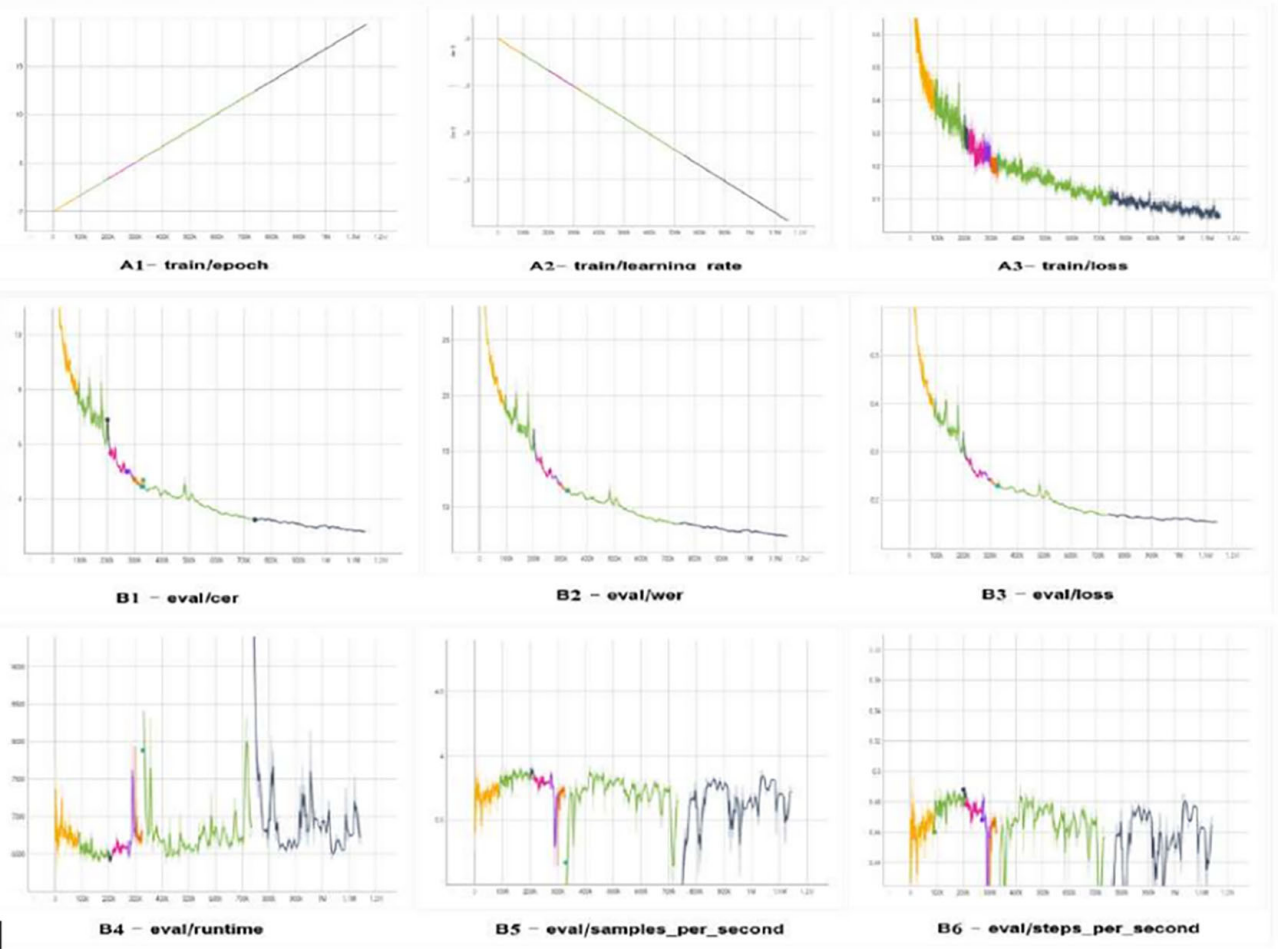
The TrOCR large model's assessment and training logs exhibit a uniform convergence with reducing loss, CER, and WER, while time and throughput hold strong despite small differences.

The learning and evaluation traces for the large version of TrOCR are displayed in Figure 12, which reveals a steady decrease in training loss, reflecting improvements at the character and word levels. The effective allocation of resources is demonstrated by the learning rate steadily decreasing within epochs, while runtime, throughput, and steps per second remain constant.

Voting, ensemble, or using a parallel decoder might enhance prediction and reduce the error rate, as shown in different research area. ^52^

The TrOCR employs cross-attention between the generative decoder and vision encoder. Advanced mechanisms such as hierarchical attention, learnable masking can be exploring in addition to semantic evaluation metrics (e.g., BERTScore) might could improve the analysis of long historical HTR sequences in future work.

### Inference

This study shows the inference which is known as “operationalizing an ML model” or “putting an ML model into production,” this procedure. Random samples were chosen for each of the three pre-trained and fine-tuned models for both Syn and human data, and most of the samples were correctly predicted, some of them were not, and the others were partially predicted.

**Figure 13.**
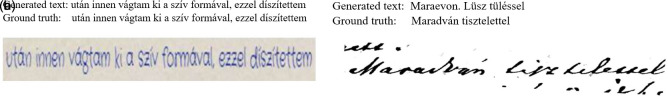
Inference for the TrOCR large model with Synthetic lines v2-1.

For the TrOCR _large-handwritten_ Pre-trained on Syn lines_hu_v2_1:

Figure 13 shows the ground truth vs. the generated text, where the first test is completely correct and the second test has some error rates.

**Figure 14.**
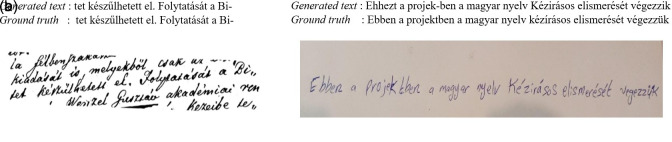
Sample of Inference for the TrOCR large model fine-tuned.

For example, in Figure 14, the prediction is correct during phase one on both pre-training and fine-tuning, while it has a false prediction when we rotate the image because the models see what humans can see.

**Figure 15.**
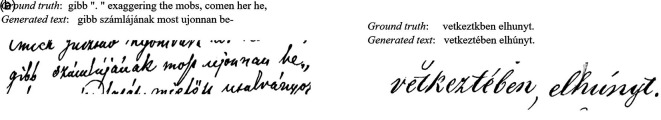
Inference on PULI- _BERT_ with Deit model fine-tuned on DH-Lab data.

The leveraged PULI BERT with the Deit checkpoint also shows a low error rate for the two-stage synthetic and human stages, as shown in Figure 15.

Roberta _base_ with Deit, Pre-train Syn lines_hu_v2_1(Stage-1)

**Figure 16.**
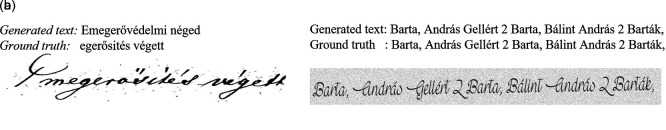
Roberta _base_ with Deit, Pre-train Syn lines_hu_v2_1 (Stage-1).

**Figure 17.**
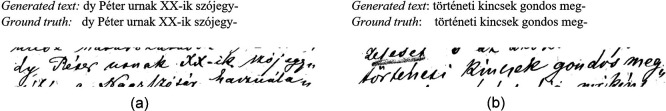
Inference for the Roberta _base_ with Deit model fine-tuned on DH-Lab (Stage-2).

The following examples are for the leveraged archaicities (Seq2Seq), where we can see the correct prediction in both Figures 16 and 17.

### Deployment

**Figure 18.**
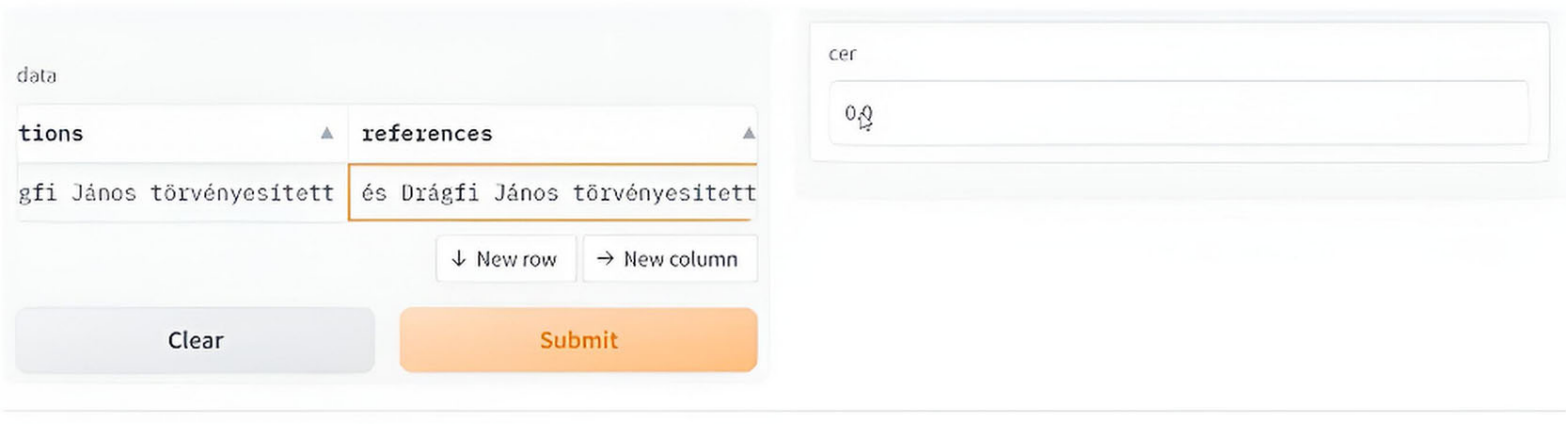
Interactive demo reference vs. Prediction.

**Figure 19.**
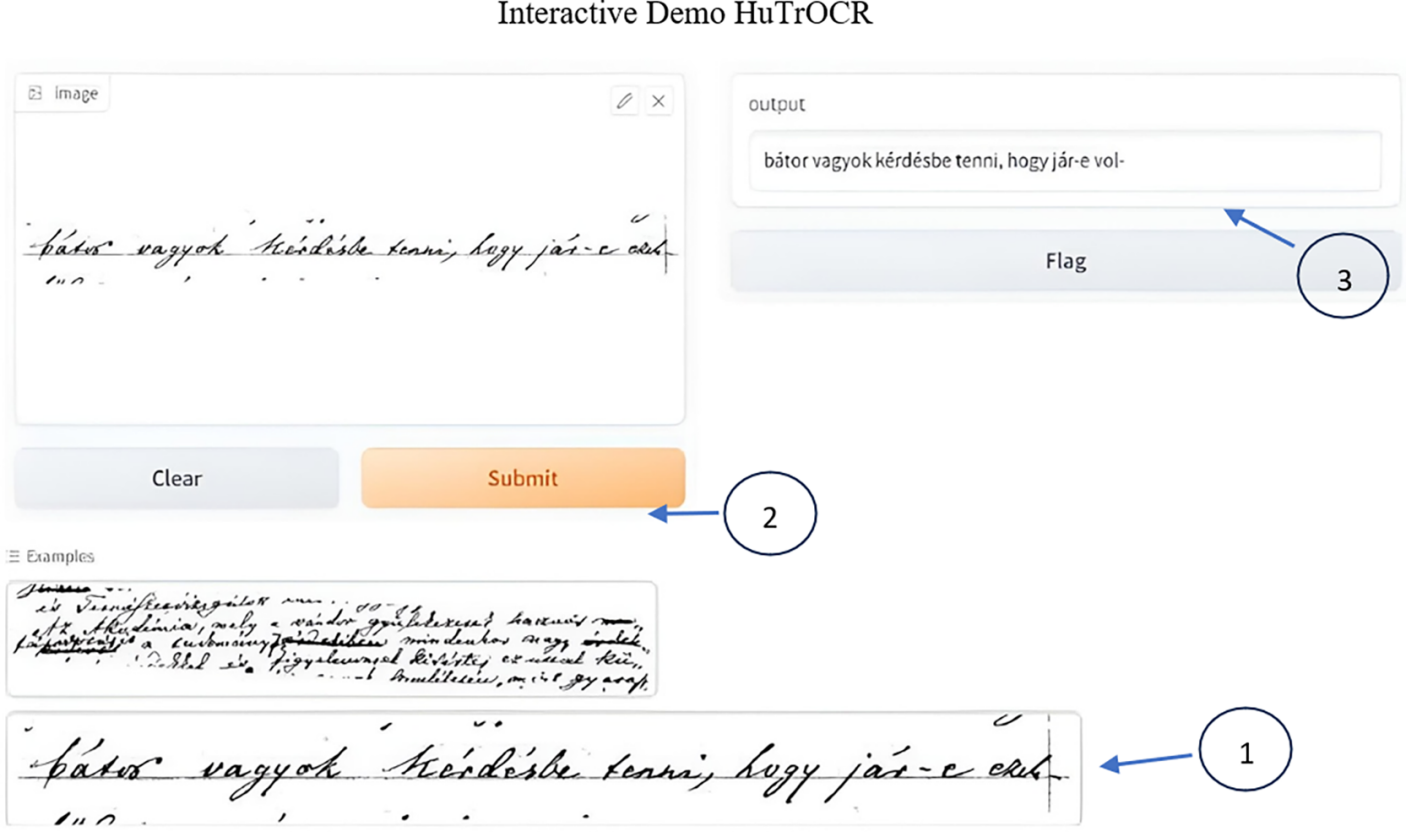
Interactive demo the user can choose from the provided samples or via upload to digitize images.

Figures 18 and 19 show a sample deployment interactive Gradio interface where the user can submit a handwritten manuscript at a line level, which is then converted to a digital format. The error rate was calculated in addition to the aforementioned evaluation metrics.

The GUI below shows the error rate (CER), which calculates the match between the reference and predicted values.

To use it first select the image or upload it, scond submit the script and you will see the resulted printed text, i.e. the ground truth for the utlized text is: “ *bátor vagyok kérdésbe tenni, hogy jár-e ezek-*”

Interactive demo HuTrOCR

Climate accountability and green living

The UN has declared global warming as an existential threat, and while discussions have been ongoing since 1972, progress has been limited. We benchmarked more than 20 training runs, of which 60% executed on NVIDIA A100 GPUs and 40% on NVIDIA Tesla T4 GPUs, for a cumulative runtime of 1690 GPU-hours. ^53^ Using nominal board powers of 400 W (A100) and 40 W (T4) and a U.S. grid carbon intensity of 387 gCO _2_/kWh, as calculated using Eq. (19), Table 8 shows that the metrics-based total energy consumption is estimated at 432.64 kWh, yielding ≈167 kg CO _2_eq. For the three principal single-GPU experiments, TrOCR (1344 h), PULI-BERT (504 h), and RoBERTa-base (504 h), totaling 2352 h, the carbon footprint ranges from ~ 36 kg CO _2_eq (all T4) to ~ 364 kg CO _2_eq (all A100), with a ~ 233 kg CO _2_eq midpoint if hours are split 60/40 between A100 and T4. This helps to streamline difficult processes, which could culminate in substantial solutions to environmental and social problems.

$$CO_{2}\left(\mathrm{kg}\right)=\left(\sum_{i}{\text{hours}}_{i}*{\text{power}}_{i}\left[\mathrm{kW}\right]\right)*\text{carbon}\_\text{intensity}\;\left[\frac{\mathrm{kg}}{\mathrm{kWh}}\right]$$ (19)

The Table 9 shows the basic standard power consumption in watt.

**Table 9.** Power consumption in watts(w).

Device (GPU)	Power(w)
Tesla T4	40
NVIDIA A100	400

## Conclusion

To sum up this study, we have successfully achieved the goals and made significant contributions to address the issue and research question: “Does the pre-training on synthetic data and fine-tuning on human data minimize the error rate?” The answer is Yes! It can be seen that the best CER is 3.681 in the TrOCR large handwritten, and the best WER is 16.091 by leveraging PULI-BERT with the Deit model with the above-mentioned enhancements. Therefore, these three models provide the best results with the methodology used and with more data, yielding better results. These fine-tuned models outperformed the current state-of-the-art TrOCR models for historical Hungarian handwriting according to the benchmark results on the János Arany dataset. During this study, a synthetic dataset was generated in addition to efficiently augmenting human data. Different levels of the experiment were performed using different transformer models and data sizes. We used word piece-based (and not character-based) methods. In conclusion, we have proven that generating synthetic data and fine-tuning human-annotated data could improve accuracy in addition to augmenting data in an efficient way, which can enhance prediction. We have seen significant improvements in the DH-Lab dataset benchmark, where the CER and WER were 5.764% and 23.297%, respectively, and have been minimized to 3.681% and 16.091%. Thus, the contribution shows the results have been improved by 2.083% and 7.206% for CER and WER, respectively. These fine-tuned models outperformed the current state-of-the-art TrOCR models for historical Hungarian handwriting according to the benchmark results on the János Arany dataset.

## Authors’ declaration

- I hereby confirm that all Figures and Tables in the manuscript are mine/ours. Furthermore, any Figures and images that are not mine/ours have been included with the necessary permission for republication, which is attached to the manuscript.

No animal studies were included in the manuscript.

No human studies were included in the manuscript.

Ethical Clearance: The project was approved by the local ethics committee at the University of Eötvös Loránd University (ELTE).

## Data Availability

The synthetic dataset generated for this study is publicly available on the Hugging Face or Zendo platform and can be accessed. If this dataset is used, please cite it as: Al-Hitawi MAS. A Synthetic Hungarian Dataset for Handwritten Text Recognition (HTR). Zenodo; 2024. https://doi.org/10.5281/zenodo.18148076. ^54^ The DH-Lab handwritten text dataset consists of human-annotated data and is not publicly available due to data protection and privacy restrictions. ^51^ Access to this dataset may be granted upon reasonable request to the corresponding author, subject to approval by the data provider.

## Footnotes

[1]
https://github.com/Mohammed20201991/OCR_HU_Tra2022

[2]
https://huggingface.co/blog/encoder-decoder

[3]
https://data.statmt.org/cc-100/hu.txt.xz, http://www.sls.hawaii.edu/bley-vroman/brown_corpus.html
