# Supplementary material for: Enhancing Transformer-Based Language Models for Hungarian Handwritten Text Recognition
Source: F1000Res. 2026 Mar 19;15:181. Originally published 2026 Feb 3. [Version 2] doi: 10.12688/f1000research.176408.2 (PMC13069383; doi:10.12688/f1000research.176408.2)
Supplement: Peer review history

# Reviewer report: Reviewer response for version 2

**DOI:** 10.5256/f1000research.197488.r469975

**Published:** 2026-04-10

**Authors:** Bal Krishna Bal

**Affiliations:** 1 Kathmandu University, Dhulikhel, Central Development Region, Nepal

The paper addresses the significant gap in offline Hungarian Handwritten Text Recognition (HTR) by introducing a robust two-stage training methodology. To overcome the scarcity of labeled data for Hungarian, the authors generated a massive synthetic dataset of 3 million text-image pairs for initial pre-training, followed by fine-tuning on the historical János Arany human-annotated dataset. Among the architectures tested—including custom Seq2Seq combinations like PULI-BERT and RoBERTa—the TrOCR large model emerged as the superior performer. It achieved a Character Error Rate (CER) of 3.681%, drastically outperforming existing benchmarks and proving that transformer-based vision-language models can effectively handle the complexities of Hungarian diacritics when supported by large-scale synthetic pre-training.

 Despite these technical achievements, the work has critical areas for refinement regarding model efficiency and generalization. A notable "augmentation paradox" was observed where data augmentation actually degraded the test performance of the TrOCR large model, suggesting potential overfitting to synthetic noise. Furthermore, while the results are impressive, the two-month training period on high-end hardware (A100 GPUs) presents a high barrier to entry, necessitating a clearer cost-benefit analysis. For the paper to reach its full potential, the authors should address minor grammatical and tabular inconsistencies, provide a qualitative error analysis of specific Hungarian characters, and clarify the terminology regarding the scale of their datasets to ensure consistency and reproducibility.

Is the work clearly and accurately presented and does it cite the current literature?

Partly

If applicable, is the statistical analysis and its interpretation appropriate?

I cannot comment. A qualified statistician is required.

Are all the source data underlying the results available to ensure full reproducibility?

Yes

Is the study design appropriate and is the work technically sound?

Yes

Are the conclusions drawn adequately supported by the results?

Yes

Are sufficient details of methods and analysis provided to allow replication by others?

Yes

Reviewer Expertise:

OCR, Script Processing, NLP, ML,DL, Text Processing

I confirm that I have read this submission and believe that I have an appropriate level of expertise to confirm that it is of an acceptable scientific standard.

# Reviewer report: Reviewer response for version 2

**DOI:** 10.5256/f1000research.197488.r469064

**Published:** 2026-03-27

**Authors:** Amjad R. Khan

**Affiliations:** 1 Prince Sultan University, Riyadh, Saudi Arabia

Accepted

Is the work clearly and accurately presented and does it cite the current literature?

Yes

If applicable, is the statistical analysis and its interpretation appropriate?

Yes

Are all the source data underlying the results available to ensure full reproducibility?

Partly

Is the study design appropriate and is the work technically sound?

Partly

Are the conclusions drawn adequately supported by the results?

Yes

Are sufficient details of methods and analysis provided to allow replication by others?

Partly

Reviewer Expertise:

IoT, Image processing, AI, Security

I confirm that I have read this submission and believe that I have an appropriate level of expertise to confirm that it is of an acceptable scientific standard.

# Reviewer report: Reviewer response for version 1

**DOI:** 10.5256/f1000research.194458.r455564

**Published:** 2026-02-16

**Authors:** Amjad R. Khan

**Affiliations:** 1 Prince Sultan University, Riyadh, Saudi Arabia

This is a well-structured, timely contribution to low-resource HTR. The methodology is sound, and the results are promising. To strengthen the paper here are major corrections required.

 Encoder diversity: The paper uses ViT/DeiT/BEiT but does not explore Swin Transformers or ConvNeXt for vision encoding, which may offer better locality modeling for handwriting.

 Decoder fine-tuning: Only BERT-family models are used as decoders. Experimenting with GPT-style autoregressive decoders or T5 might yield different trade-offs in generative quality.

 Attention mechanisms: Consider incorporating cross-attention with learnable masking or hierarchical attention to better handle long handwritten lines.

 For historical documents, semantic similarity metrics (e.g., BERTScore) or word/sentence embeddings similarity could provide deeper insight into meaningful errors.

 Error analysis: A breakdown of errors by character type (diacritics, ligatures) or word frequency would help identify specific weaknesses.

 Typos: “ch has been successful” (Page 8) seems incomplete.

 Figure references: Some figures are referenced but not included in the provided text (e.g., Fig. 2, 3, 5–7, 11–19).

 Equations: Eq. (9) formatting is unclear. Consider renumbering or clarifying.

 Comparisons with existing methods are limited and do not adequately represent recent state-of-the-art approaches.

 Saifullah, Aslam, M., Martinez-Enriquez, A. M., & Khan, M. U. G. (2025). A Blockchain Based Intelligent System for Urdu Information Veracity Assessment. International Journal of Theoretical & Applied Computational Intelligence, vol. 2025, 284–304. https://doi.org/10.65278/IJTACI.2025.27

 Saba, T., Rehman, A., & Sulong, G. (2011). Cursive script segmentation with neural confidence. Int J Innov Comput Inf Control (IJICIC), 7(7), 1-10.

 Rehman, A., & Saba, T. (2012). Off-line cursive script recognition: current advances, comparisons and remaining problems. Artificial Intelligence Review, 37(4), 261-288.

 Saba, T., Rehman, A., & Elarbi-Boudihir, M. (2014). Methods and strategies on off-line cursive touched characters segmentation: a directional review. Artificial Intelligence Review, 42(4), 1047-1066.

 Ali, M. H., & Rasheed, M. A. (2025). A Blockchain-Based Multi-Agent Security Framework for E-Commerce Systems. International Journal of Theoretical & Applied Computational Intelligence, vol. 2025, 227–245. https://doi.org/10.65278/IJTACI.2025.15

Is the work clearly and accurately presented and does it cite the current literature?

Yes

If applicable, is the statistical analysis and its interpretation appropriate?

Yes

Are all the source data underlying the results available to ensure full reproducibility?

Partly

Is the study design appropriate and is the work technically sound?

Partly

Are the conclusions drawn adequately supported by the results?

Yes

Are sufficient details of methods and analysis provided to allow replication by others?

Partly

Reviewer Expertise:

IoT, Image processing, AI, Security

I confirm that I have read this submission and believe that I have an appropriate level of expertise to confirm that it is of an acceptable scientific standard, however I have significant reservations, as outlined above.

# Response

**Published:** 2026-03-06

**Authors:** Mohammed Al-Hitawi

**Affiliations:** Artificial Intelligence, College of Information Technology, University of Fallujah, Fallujah, Anbar, Iraq

We sincerely thank the reviewer for the careful evaluation of our manuscript and for the constructive comments that helped improve the quality and clarity of this work which is available in V2.

- Answer-1:
  We evaluated the Swin Transformer as a visual encoder within the TrOCR framework using the same training configuration. The results showed performance comparable to Vision Transformer, DeiT, and BEiT, with slightly higher CER/WER values. Since the differences were minor, these results were not included in the manuscript to maintain conciseness, but (now included in a v2) the full experiment is available in our GitHub repository.
- Answer-2: 
  Preliminary experiments with GPT-2 and multilingual GPT variants within the TrOCR framework showed unstable convergence and significantly higher CER/WER compared with BERT decoders. The experimental results are available in the project repository, and a clarification has been added to the revised manuscript.
- Answer-3: 
  The employed TrOCR model already utilizes cross-attention between the visual encoder and text decoder. However, more advanced mechanisms such as hierarchical attention or learnable masking were beyond the scope of this work. Additionally, while evaluation was performed using CER and WER, semantic metrics such as BERTScore could provide deeper insight into recognition errors in historical texts. These aspects have been noted as potential directions for future work and clarified in the revised manuscript.
- Answer-4: 
  The current study reports overall performance using Character Error Rate and Word Error Rate. A detailed error breakdown by character type (e.g., diacritics or ligatures) and word frequency analysis is valuable and has been noted as future work in the revised manuscript(end of Evaluation Metrics).
- Answer-5: 
  The sentence has been corrected in the revised manuscript.
- Answer-6: 
  All referenced figures (e.g., Fig. 2, 3, 5–7, 11–19) are included in the manuscript. The revised version has been carefully checked to ensure that all figure references correctly correspond to the included figures.
- Answer-7: 
  The formatting of Eq. (9) has been revised and clarified in the manuscript to improve readability and consistency with Eq. (8).
- Answer-8: 
  The related work section has been expanded to include segmentation-based handwriting recognition studies, such as the work of Tariq Saba and Abdul Rehman[53]. The manuscript now clarifies the difference between these approaches and the transformer-based method used in this study. Additionally Table 8 updated for comparison with existing studies [54]. 
